# Kearns-Sayre Syndrome With Persistent Ventricular Tachycardia Refractory to Shocks and Medications

**DOI:** 10.7759/cureus.17175

**Published:** 2021-08-14

**Authors:** Ndausung Udongwo, Dhairya Gor, Kyle Wiseman, Abbas Alshami, Steven Daniels

**Affiliations:** 1 Internal Medicine, Jersey Shore University Medical Center, Neptune, USA; 2 Cardiology, Jersey Shore University Medical Center, Neptune, USA

**Keywords:** kearns-sayre syndrome, ventricular tachycardia storm, arrhythmias, sudden cardiac death, heart blocks

## Abstract

Cardiovascular conduction delay makes up part of the triad associated with Kearns-Sayre syndrome (KSS). Although there have been a few reported cases of prolonged Qtc and polymorphic ventricular tachycardia associated with this disease, despite the use of automatic implantable cardioverter defibrillators (AICD) for secondary prevention, some cases have been reported where the use of AICD did not help. We present a case of a 62-year-old male with KSS who came to the emergency department (ED) after two episodes of syncope. He already had an automatic AICD placed at the age of 34. Our patient had Qtc prolongation which is an unusual finding in KSS. He also had recurrent ventricular tachycardia (VT) refractory to medications and multiple shocks from his AICD, which progressed to a VT storm. He eventually passed away after the withdrawal of care, as his prognosis worsened. We recommend that a more clear guideline will help manage this devastating disease, resulting in mortality reduction.

## Introduction

Kearns-Sayre syndrome (KSS) is a rare mitochondrial disorder often accompanied by muscle weakness and has a reported prevalence of 0.001%-0.003% worldwide [1-2]. It was first described in 1958 [2]. This syndrome usually occurs before the age of 20 and often presents with the triad of ophthalmoplegia, heart block, and pigmentary retinopathy [1-3]. Its cardiac involvement is reportedly common and can lead to fatal arrhythmias with sudden cardiac death [2]. Several clues found on an electrocardiogram (EKG) can be a life-saving indicator of early conduction system disturbances, leading to earlier intervention and overall improved outcomes [3]. Some of these clues include a prolonged QTc, potentially leading to sustained ventricular tachycardia (VT) and even VT storm, which refers to three or more episodes of sustained VT within a 24-hour period or incessant VT for at least 12 hours [4-5]. We present a case of a 62-year-old male with a history of KSS associated with VT with progression to VT storm despite being on an AICD. To the best of our knowledge, there are only a few reported cases of refractory VT storms associated with this syndrome.

## Case presentation

A 62-year-old male with a past medical history of Kearns-Sayre syndrome (KSS) with multi-system involvement including skeletal muscle myopathy with chronic progressive ophthalmoplegia, pigmentary retinopathy, heart failure (HF) with reduced ejection fraction (11%-15%), non-sustained ventricular tachycardia, pulmonary hypertension, chronic heart block with biventricular automatic implantable cardioverter defibrillators (AICD), chronic kidney disease, hypertension, hyperlipidemia, and mitral valve prolapse presented to the emergency department (ED) after two syncopal episodes.

He received two shocks from the device, one for each episode. He denied any chest pain, palpitations, dyspnea at rest, diaphoresis, nausea, or vomiting. Interrogation of the AICD showed two appropriate shocks after failed anti-tachycardia pacing (ATP). Home medications were mexiletine 300 mg three times daily, sotalol 80 mg twice daily, hydralazine 10 mg three times daily, carvedilol 3.125 mg twice daily, furosemide 20 mg once daily, and milrinone 0.5 mcg/kg/min intravenously through his peripherally inserted central catheter (PICC) line. He was allergic to ramipril and valsartan. He denied any tobacco or alcohol use in the past. Family history was remarkable for KSS in two of his sisters, with one who had passed away from a decompensated HF. 

On physical examination, vitals showed a blood pressure of 110/73 mmHg, heart rate of 81 beats per minute, temperature of 97.5°F, respiratory rate of 16 breaths per minute (bpm), and oxygen saturation of 98% on room air. He looked cachectic with a body mass index (BMI) of 15 kg/m2. He had significant left-sided ptosis. A cardiac exam revealed a grade II/VI systolic murmur but no gallop. A pulmonary exam revealed decreased breath sounds in the right lower lung field. He was admitted for syncope secondary to VT, 150 mg of IV amiodarone bolus was administered, and then he was started on amiodarone drip at 0.5 mg/min.

The laboratory results are shown in Table 1. 

**Table 1:** Summary of initial laboratory results.

Blood	Result	References
Hemoglobin (g/dL)	13.4	12.0-16.0 (g/dL)
White blood cells (10*3u/L)	6.0	4.5-11.0 (10*3u/L)
Glucose (mg/dL)	114	70-99 (mg/dL)
Creatinine (mg/dL)	2.01	0.61-1.24 (mg/dL)
Sodium (mmol/L)	137	136-146 (mmol/L)
Potassium (mmol/L)	5	3.5-5.0 (mmol/L)
Bicarbonate (mmol/L)	28	7-18 (mmol/L)
Magnesium (mg/dL)	2	1.3-2.5 (mg/dL)
Troponin (ng/mL)	<0.04	<0.04 ng/mL
B-Type natriuretic peptide (BNP) (pg/mL)	3,050	<= 100 pg/mL

**Figure 1:**
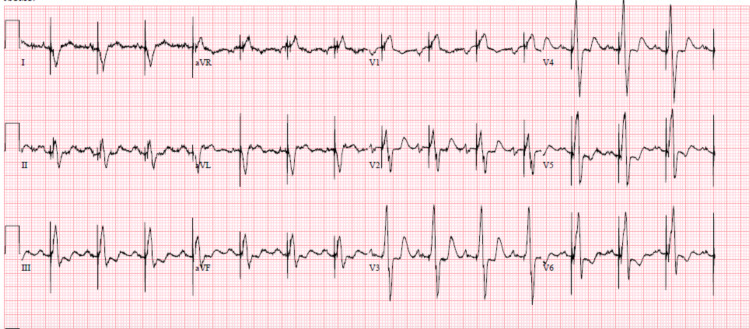
Initial electrocardiogram.

Electrocardiogram results showed; atrial sensed, and a ventricular paced rhythm at 86 bpm with no ST or T wave changes, and a Qtc of 564 milliseconds (Figure 1).

**Figure 2:**
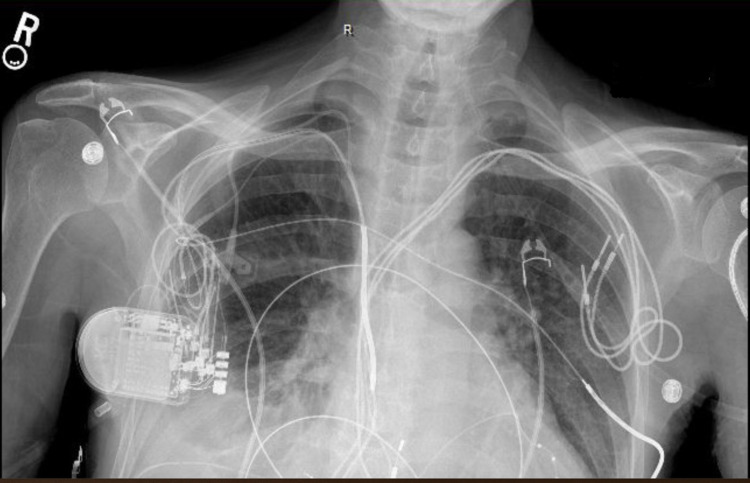
Chest X-ray showing right pleural effusion with adjacent atelectasis.

Further investigation of the AICD yielded sustained monomorphic ventricular tachycardia to the 140s during both syncopal episodes. Chest X-ray revealed right pleural effusion (Figure 2).

A CT scan of the head was unremarkable. 

On day two, the patient was noted to have non-sustained VT with increasing oxygen requirements. A plan was in place to transition the patient from IV to PO amiodarone, but instead, he went into VT storm and was thus continued on amiodarone drip at 0.5 mg/min. The AICD yielded ineffective anti-tachycardia pacing (ATP) and two shocks, the first of which failed. He was transferred to the critical care unit (CCU) for further monitoring and started on lidocaine drip (1 mg/min) with continued amiodarone and milrinone drips (0.5 mg/min and 0.375 mcg/kg/min respectively).

In the CCU, he continued to have non-sustained VT with symptoms of palpitations despite the AICD reprogramming and medical management (antiarrhythmic medications and electrolyte repletion). He also continued to have shortness of breath with minimal improvement on bilevel positive airway pressure (BIPAP). A nephrologist was consulted as his renal function began to worsen. Unfortunately, recurrent VT progressed to a VT storm, requiring multiple boluses of IV amiodarone (150 mg), IV lidocaine (100 mg), and multiple shocks from his AICD which were futile. The VT storm was refractory to his current medical management. The patient eventually passed away after the withdrawal of care. 

## Discussion

Kearns-Sayre syndrome is a progressive disease that leads to sudden cardiac death in up to 20% of the cases reported, thus warranting definitive guidance to detect and treat potential underlying cardiac abnormalities that may precipitate such an event [1]. Our patient had KSS with a family history of sudden cardiac death (SCD) from the same syndrome. He presented for a syncope workup and was found to have recurrent VT, progressing to VT storm despite AICD reprogramming, antiarrhythmic medications, and electrolyte repletion. Although electrical storms (VT storms) occur in patients with low left ventricular ejection fractions and structural heart disease, inherited syndromes causing arrhythmias (KSS, Brugada) predispose patients to this debilitating arrhythmia [2-3]. We believe that our patient’s recurrent VT storm that was refractory to medical/electrical management was due to KSS. 

Generally, the first cardiac manifestation in KSS is an atrioventricular block [1, 3-7]. Although it can progress from a bundle branch block to a complete heart block, the succession is usually unpredictable [1-2]. Less commonly, it is also linked to ventricular arrhythmias [8]. The most common type of ventricular arrhythmia seen is polymorphic ventricular tachycardia [3]. There are also reports of torsade de pointes and ventricular fibrillation in these patients [6]. Although the risk of sudden death is mainly assumed to be due to complete heart block, ventricular arrhythmias should also be acknowledged [6]. Our patient was having episodes of asymptomatic ventricular tachycardia up until his passing, with a brief episode of torsades. 

In the pathophysiology of KSS-induced arrhythmia, there is mitochondrial dysfunction leading to increased intracellular calcium concentration with delayed after-depolarizations (DADs). Bradycardia caused by the A-V block has been shown to induce early afterdepolarizations (EADs) in a systematic review article published by Imamura et al. [4]. Pacemakers and antiarrhythmic medications such as mexiletine can successfully suppress EADs, but patients remain at risk for fatal ventricular arrhythmias [4]. Furthermore, the association of KSS and QT prolongation is not well established. Regardless, such high-risk patients with prolonged QT can quickly develop ventricular arrhythmias and should be identified early [6-8]. Our patient’s EKG during his hospital stay was notable for a QTc of 564 milliseconds, which was likely a contributing factor to his VT. His prolonged QTc may have also been due to his sotalol use in the setting of renal insufficiency. Studies have shown that patients with a normal functioning pacemaker can have a QT prolongation, precipitating a ventricular arrhythmia. Ventricular arrhythmia is also described in the absence of QT prolongation or bradycardia [9]. However, structural abnormalities were not ruled out in such cases [9-10]. Also, whether patients with QT prolongation should have a defibrillator placed is an attractive question to consider [8]. The current American Heart Association/American College of Cardiology/Heart Rhythm Society (AHA/ACC/HRS) guidelines recommend PPM implantation [class1, level b] for advanced second- or third-degree blocks with or without symptoms in KSS [9]. However, it is suggested that pacemakers may not be enough, and ICD placements should be considered [6, 8]; however, no official recommendations yet exist. 

Very few cases have been reported where an ICD patient with KSS experienced a fatal ventricular arrhythmia. Our patient had an ICD placed at the age of 34, which ameliorated many episodes of VT as his disease slowly progressed. Imamura et al. presented a patient with KSS complicated by multiple hospital admissions for VT, torsades de pointes, and ventricular fibrillation who was given an ICD, followed by resolution arrhythmias and marked improvement in her quality of life [4]. Unfortunately, our case differs as our patient’s ventricular arrhythmia persisted until he passed away. Our case is rare, and we suggest that further guidelines be addressed on the management of KSS, emphasizing the prevention of fatal cardiac arrhythmias.

## Conclusions

Sudden cardiac death in KSS is often attributed to the heart block brought on by cardiac myocyte changes early in the disease. However, ventricular arrhythmias, including VT storms, can also be an additional cause of mortality. Evidence of myocyte ion channel abnormalities such as prolonged QTc suggests the importance of considering AICD implantation early before the onset of ventricular arrhythmias. Prolonged Qtc in patients with KSS is an unusual finding and poses an increased risk for SCD. We recommend that new guidelines be made to help in the management of the fatal cardiac arrhythmias that are associated with this syndrome. 
